# Epigenetic opportunities for evolutionary computation

**DOI:** 10.1098/rsos.221256

**Published:** 2023-05-10

**Authors:** Sizhe Yuen, Thomas H. G. Ezard, Adam J. Sobey

**Affiliations:** ^1^ Maritime Engineering, University of Southampton, Southampton SO17 1BJ, UK; ^2^ Ocean and Earth Science, National Oceanography Centre Southampton, European Way, University of Southampton, Southampton SO14 3ZH, UK; ^3^ Marine and Maritime Group, Data-centric Engineering, The Alan Turing Institute, The British Library, London NW1 2DB, UK

**Keywords:** evolutionary algorithms, evolutionary computation, swarm intelligence, evolutionary biology, non-genetic inheritance, epigenetics

## Abstract

Evolutionary computation is a group of biologically inspired algorithms used to solve complex optimization problems. It can be split into evolutionary algorithms, which take inspiration from genetic inheritance, and swarm intelligence algorithms, that take inspiration from cultural inheritance. However, much of the modern evolutionary literature remains relatively unexplored. To understand which evolutionary mechanisms have been considered, and which have been overlooked, this paper breaks down successful bioinspired algorithms under a contemporary biological framework based on the extended evolutionary synthesis, an extension of the classical, genetics focused, modern synthesis. Although the idea of the extended evolutionary synthesis has not been fully accepted in evolutionary theory, it presents many interesting concepts that could provide benefits to evolutionary computation. The analysis shows that Darwinism and the modern synthesis have been incorporated into evolutionary computation but the extended evolutionary synthesis has been broadly ignored beyond: cultural inheritance, incorporated in the sub-set of swarm intelligence algorithms, evolvability, through covariance matrix adaptation evolution strategy (CMA-ES), and multilevel selection, through multilevel selection genetic algorithm (MLSGA). The framework shows a gap in epigenetic inheritance for evolutionary computation, despite being a key building block in modern interpretations of evolution. This leaves a diverse range of biologically inspired mechanisms as low hanging fruit that should be explored further within evolutionary computation and illustrates the potential of epigenetic based approaches through the recent benchmarks in the literature.

## 1. Maximizing the potential from biological analogies

Evolutionary computation has emerged as one of the most studied branches of Artificial Intelligence. Hundreds of approaches have been reported over the years, based on different bioinspired behaviours. However, it is difficult to determine the uniqueness of each algorithm when new approaches only change the vocabulary and not the underlying mathematics. The constant addition of new algorithms has led to discussions about whether the biological analogies have gone too far [1] with some arguing that chasing new novel metaphors for algorithms risks moving attention away from innovative ideas that make a real difference. However, new genetic algorithms, such as coevolutionary multilevel selection genetic algorithm (cMLSGA) [2], have successfully explored additional non-genetic or indirectly genetic mechanisms, and found benefits that improve performance on a growing number of practical problems [3,4]. The key to success for evolutionary computation appears to be exploration of new mechanisms, bioinspired or not, without repeatedly exploring the same mechanisms under a new name.

**Figure 1.**
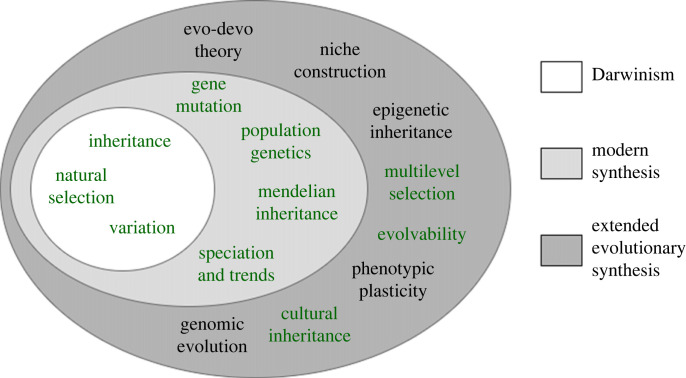
Key concepts of Darwinism, the modern synthesis and the extended evolutionary synthesis. Highlighted concepts are currently explored in evolutionary computation and swarm intelligence algorithms.

In evolutionary theory, the modern synthesis [5] was developed throughout the first half of the twentieth century to combine the ideas of Darwin and Wallace’s evolution by natural selection [6,7], and Mendel’s principles of inheritance [8]. Despite the contribution to evolution from these mechanisms, there is growing evidence suggesting that non-genetic inheritance also has a strong effect. New research into the concepts of non-genetic inheritance suggests that the ideas of the modern synthesis should be extended [9,10] to include the effects of epigenetics, cultural inheritance, phenotypic plasticity, parental and environmental effects and multilevel selection. However, despite the number of algorithms in evolutionary computation, the focus is on a relatively small part of evolutionary theory with most evolutionary algorithms directly comparable to the concepts of the modern synthesis and swarm intelligence compared directly to cultural inheritance. Figure 1 shows the concepts of the modern synthesis and the extended evolutionary synthesis, and highlights the concepts which have been explored. While the genetic concepts of Darwinism and the modern synthesis have been studied in detail, only a few concepts from the extended evolutionary synthesis have been explored within evolutionary computation, with only limited efforts to bring these other non-genetic inheritance concepts to the field.

However, there is a growing literature showing that there are benefits to investigating the outstanding evolutionary concepts not routinely included in evolutionary algorithms, as demonstrated by CMA-ES and cMLSGA. Investigating other processes from the extended evolutionary synthesis is important to push the field forward with mechanisms that have been shown to provide novel differences in evolutionary biology. The problem is the large number of algorithms developed each year, each pointing to novel mechanisms but which from an evolutionary perspective have limited differences. To help make distinctions between different novel algorithms it is important to understand the differences in their mechanisms, which can be aided through a better understanding of the underlying biological concepts and how close the new algorithms are to mimicry or inspiration. What are the biological roots and operators used? What are the key similarities and mathematical differences between two bioinspired algorithms apart from the terminology used?

First, this paper introduces the concepts of the extended evolutionary synthesis and their benefits in nature. Then, to help ensure that future bioinspired algorithms do not recycle the same concepts, it categorizes the current state of the art in evolutionary computation under a biological framework. The framework can show how current algorithms relate to each other and demonstrate gaps in the evolutionary synthesis that computational algorithms have yet to explore. Details of each algorithm are then discussed and it is shown how their mechanisms are similar to each other. Finally, epigenetics are highlighted as the most promising of the remaining mechanisms available and recent advances in epigenetic evolutionary computation are discussed.

## 2. Moving towards an extended evolutionary synthesis

The elements of the extended evolutionary synthesis expand on the existing concepts of the modern synthesis to allow for a broader range of ideas and explanations [9,10]. These concepts give further explanations for how biological organisms can adapt and change to the environment quickly compared to natural selection, that can’t be explained purely through the original concept developed by Mendel and Darwin. In the modern synthesis, phenotypic variation is seen as random based on genetic variation and inheritance. Natural selection then applies the environmental pressure to determine the fitness of different individuals and species. In the extended evolutionary synthesis, it is argued that phenotypic variation can be guided rather than random, allowing organisms to combat selection pressures with fast adaptive variations to prevailing environmental conditions. For example, evolutionary developmental biology (evo-devo) bridges the gap between developmental biology and evolutionary biology [11], providing an understanding of how individual development occurs, and how the developmental process directs phenotypic variation in individuals.

### 2.1. Relating current bioinspired algorithms to the modern extended evolutionary synthesis

In evolutionary computation, the literature is divided between two general fields:

— **Evolutionary algorithms** [12] (EA)—Algorithms with a foundation in genetics, these date back to Turing’s learning machine [13], and include the branches of evolutionary programming (EP), evolution strategies (ES), and genetic algorithms (GA). These algorithms generally involve the genetic mechanisms of selection, recombination (crossover), and mutation.
— **Swarm intelligence** [14] (SI)—Algorithms based on collective intelligence with patterns of communication and interaction in a population. Swarm intelligence algorithms cover a wide range of biological inspirations, from animal behaviour algorithms such as particle swarm optimization [15] (PSO) and ant colony optimization [16] (ACO), as well as more esoteric inspirations such as political anarchy (Anarchic Society Optimization [17]).

In evolutionary algorithms, individuals are presented as a genotype that evolves with genetic operators. The algorithms use genetic operators to represent candidate solutions as genotypes and the genetic mechanisms evolve the genotype through genetic inheritance. Conversely, individuals in swarm intelligence algorithms are typically presented as phenotypes, which are an organism’s observable traits such as physical appearance or behaviour. Phenotypes are the target for selection, for example bigger horns, whereas genotypes represent the response to selection. These phenotypes are often used in metaphors to hunting, foraging, and movement behaviour, with few genetic components. The focus of the extended evolutionary synthesis is that there are many interacting routes that influence the final phenotype, not only genetic inheritance, as encoded in evolutionary algorithms, or within-generation cultural transmission, as encoded in swarm intelligence, but rather a mix of influences both within and across generations. This diversity of influences reduces reliance on a single mode of inheritance and generates phenotypes from a diversified portfolio of influences This allows hedging against maladaptations while also providing more rapid adaptation when genetic, indirect genetic and phenotypic effects align [10].

**Figure 2.**
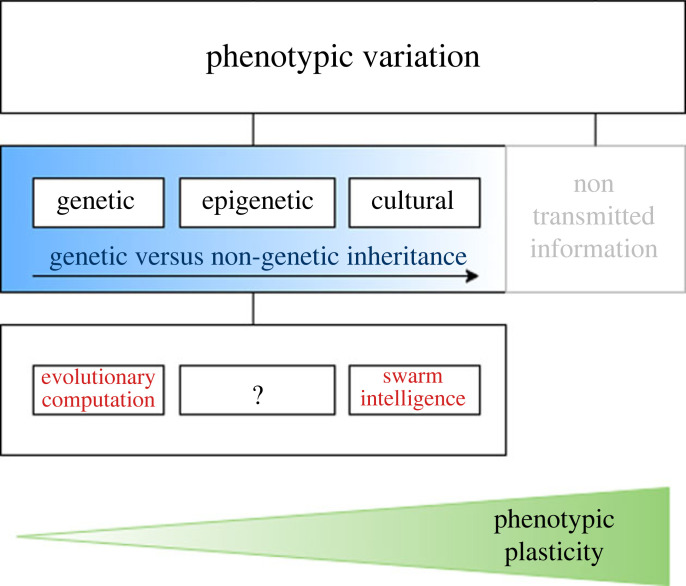
Sources of information transmission for phenotypic variation linked to inspiration for bioinspired algorithms adapted from [18].

Using a biological framework for information transmission [18], figure 2 shows how the genetic and cultural sources of information are closely linked to evolutionary algorithms and swarm intelligence, respectively. The mechanisms further right leverage non-genetic transmission more than those on the left. Evolutionary algorithms are mostly based on the genetic source while swarm intelligence methods are based on the cultural source. Phenotypic plasticity is the ability of the same genotype to produce different phenotypes in response to epigenetic or environmental conditions [19]. On the far left, genetic inheritance has low plasticity as genotypes take many generations to mutate and evolve. Genetic inheritance by itself cannot react to sudden changes to the environment and adapt the genotype immediately. Cultural and non-transmitted information can lead to higher phenotypic plasticity and higher variation, as in nature as adaptation can occur quickly within a few generations. On the far right, non-transmitted information is information that is not inherited by future generations; this information has the highest plasticity as it can act immediately on environmental changes, but is unstable and does not carry forward to future generations.

Non-transmitted information is included in some algorithms that use a population of individuals acting with a certain behaviour that does not require interaction with other individuals. For example, the Bat Algorithm [20] is inspired by the echolocation techniques of bats. The individual bats interact with the environment to locate prey, but do not transmit information with each other. The mechanisms for each mode of transmission are further documented in table 1.

**Table 1.** Biological categories of transmission for different levels of evolution.

category	sub-category	description
genetic	selection	In each generation, a subset of the population is chosen to reproduce the next generation. In biological terms, this relates to the survival of the fittest. Individuals with fitter genes are typically able to live longer and produce more offspring. In computational terms, individuals are selected based on their fitness against the objective function.
	recombination	Also called crossover, when two individuals produce offspring, genetic material from both parents are used. This can be in a sexual or asexual way. This results in offspring that share some attributes with both parents. Crossover affects both diversity and convergence of the population as it creates new combinations while keeping the same base genetic material from its parents, allowing fit genes to continue propagating through multiple generations in the population.
	mutation	When offspring are formed, mutation can occur. These mutations could be neutral, beneficial or detrimental to the survival of that individual. Mutation helps improve diversity by producing genetic material that has not been explored before by the population.
epigenetic	mitotic	The transmission of epigenetic changes through mitotic cell divisions. Epigenetic marks cause variations to occur in an individual which are propagated through cell divisions when the marks are triggered as a response to environmental cues. Mitotic epigenetic inheritance only transfers the epigenetic changes across the same generation.
	germline	Epigenetic changes caused by environmental factors in the parent are passed down to the offspring and to further generations [21]. This affects individuals across multiple generations, even if the environmental factor that triggered the change only happened during the first generation.
	experience-dependent	Epigenetic marks that influence parental behaviour causing the same epigenetic mark to appear in the offspring generation. The marks can persist across multiple generations, but the transmission can also be broken when environmental factors cause one generation to stop the same parental behaviour. For example, maternal behaviour in rodents cause the offspring to exhibit the same behaviour as their offspring. But if there is a break in one generation where the maternal behaviour does not occur, the epigenetic transmission stops [22].
cultural	specialized roles	Individuals in a population have different roles to fulfil. For example, scout and guard bees in a colony, or mongooses that form foraging niches [23] due to competition within the population. In some cases, specialized roles are formed and have lasted through millions of generations, as in the case of ants and bees. In others such as the mongooses, the specialized roles may only form in a particular generation due to external factors during the generation’s lifespan.
	social learning (individual level)	Individuals learning from other individuals in the population through direct information sharing, teaching, environmental stimulus or imitation and emulation of other individuals.
	social learning (population level)	Information transfer between populations (cultures) where different populations may have different variations in behaviour as a result of social learning.

## 3. Categorizing algorithms

Table 2 breaks down each category of inheritance into the biological concepts defined in table 1, to show the overlap between the selected algorithms. Due to the number of algorithms in the available literature, a representative set were chosen based on if they were popular within the evolutionary algorithm and swarm intelligence fields, defined as more than 1000 citations, or if they showed strong performance in recent benchmarking studies (appendix A). There is a split between evolutionary algorithms, which all use selection, recombination, and mutation operators, and swarm intelligence algorithms, which have a mix between specialized roles for individuals and different forms of social learning and communication. While some algorithms fit neatly into genetic or cultural information sharing, contrasting with evolutionary theory where multiple mechanisms act together, there are a number of algorithms that use a spread of information mechanisms: cMLSGA uses specialized roles and social learning, while GB-ABC and Firefly Algorithm (FA) use elements of selection; GB-ABC and EBO/CMAR use recombination; HEIA uses coevolution to spread social learning; and Cuckoo Search uses genetically modified nests with crossover and mutation operators. The introduction of multiple mechanisms has recently proven to balance convergence and diversity, with cMLSGA and HEIA showing top performance on state-of-the-art multi-objective benchmarking problems [2], and EBO/CMAR shows top performance in a CEC’17 single objective bound constrained competition [24], an area normally dominated by variants of GAs and DE algorithms. Some other algorithms such as the univariate marginal distribution algorithm (UMDA) [25] and compact genetic algorithm (cGA) [26] fall under the category of estimation of distribution algorithms (EGAs), which do not fit into the same biological categorizations as the other evolutionary algorithms and swarm intelligence algorithms as they use explicit probability distribution models over biological mechanisms. While some existing studies [27–29] investigate the use of epigenetics in evolutionary computation, neither their performance nor popularity fit the criteria to be included in this discussion. It is proposed that this is because they do not capture the inheritance and transfer of epigenetic information to future generations, and so miss out the adaptability and reversibility of epigenetics. A discussion on the key concepts of epigenetics and why these studies miss these concepts is presented in §5. A justification of table 2 is given in the following subsections.

**Table 2.** How bio-inspired algorithms fit into a biological framework for different forms of inheritance.

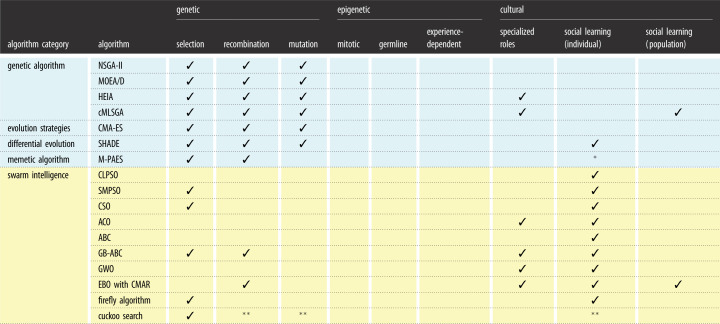

*Memetic algorithm variants which use PSOs as their local search operators include the PSO cultural mechanism of social learning.

**Cuckoo Search variants (Cuckoo-GRN and Modified Cuckoo Search) include additional genetic and cultural mechanisms, respectively.

NSGA-II, non-dominated sorting genetic algorithm II; MOEA/D, multiobjective evolutionary algorithm based on decomposition; HEIA, hybrid evolutionary immune algorithm; cMLSGA, coevolutionary multilevel selection genetic algorithm; CMA-ES, covariance matrix adaptation evolution strategy; SHADE, success-history based parameter adaptation for differential evolution; M-PAES: memetic pareto archive evolution strategy; CLPSO, comprehensive learning particle swarm optimization; SMPSO, speed-constrained multi-objective particle swarm optimization; CSO, competitive Swarm optimizer; ACO, ant colony optimization; ABC, artificial bee colony; GB-ABC, artificial bee colony algorithm based on genetic operators; GWO, Grey Wolf Optimizer; EBO with CMAR, effective butterfly optimizer with covariance matrix adapted retreat phase.

### 3.1. Genetic algorithms

GA [30] are inspired by natural selection, evolving a population of potential solutions towards an optimal solution. They are tightly linked to a simplified concept of genetic evolution using selection, crossover and mutation mechanics. The focus in development is almost entirely related to different selection mechanisms, with only a small number of modern crossover and mutation mechanisms preferred, which are no longer bioinspired. In addition to these operators, many GAs also incorporate other mechanics such as elitism [31] as a representation of ‘survival of the fittest’; which keeps the best individuals in a population, copying them directly into the next generation to improve convergence at the cost of diversity. This provides a considerable computational benefit and ensures that the fittest solution can’t be lost over the generations.

#### 3.1.1. Niching GAs

The NSGA-II [32] algorithm belongs to a family of niching algorithms, where the most popular versions are NSGA-II and its refinement NSGA-III [33], which is an extension for many-objective problems. The difference between this approach and other GA is the use of non-dominated sorting to rank individuals for selection but it also uses a density estimator to maintain diversity in the population. The density estimation is not a genetic operator, as it does not involve the genotype of candidate solutions; it is not epigenetic as it is not inherited between individuals, and it is not cultural as it is not learned through interaction between individuals, rather it is forced upon the population as an external system to ensure individuals are spread out along the Pareto front. As such it uses computational elements to improve selection but still incorporates selection, recombination and mutation.

#### 3.1.2. Decomposition based GAs

MOEA/D [34] is a GA approach that uses mathematical decomposition to split a multi-objective problem into a number of sub-problems with an assigned weight vector. The population is split to solve each sub-problem separately, where each individual can only reproduce with individuals within the same neighbourhood. Although the population is divided into sub-populations and reproduction between neighbourhoods of sub-populations is allowed, there is no learning or interaction between individuals for social learning and this boosts exploitation of the search but reduces the exploration. Many variants of the original MOEA/D algorithm focus on improving different aspects of the original algorithm such as the decomposition method [35,36] or weight vector generation [37,38]. Compared to other approaches the development of MOEA/D focuses on mathematical methods for selection but still incorporates selection, recombination, and mutation.

#### 3.1.3. Coevolutionary GAs

Coevolutionary GAs use cooperation or competition between two populations or algorithms to find the best solutions. These have been gaining more popularity in the evolutionary computation literature, with papers proposing that coevolution creates a generalist approach which reduces hyperparamter tuning [4]. The top performing method is a hybrid coevolutionary method HEIA [39] that also uses sub-populations, but instead of dividing based on decomposition of the problem, the sub-populations use different algorithms for evolution, representing different selective regimes. After each generation, the best individuals from each sub-population are saved in an external archive and the sub-populations are cloned for the next generation with an immune algorithm. With this coevolutionary approach, the sub-populations can naturally develop specialized roles to solve the problem. This means the algorithm incorporates selection, recombination, mutation and specialized roles, but with a focus on exploration of the space.

#### 3.1.4. Multilevel selection

Multilevel selection is the idea that natural selection occurs at different levels such as the genetic level [40], the individual organism level [41] or the species level. At the genetic level, selection occurs on individual genes through recombination, where genes may act selfishly and optimize for selection to the next generations, potentially at the cost of the total fitness of an organism. The existence of altruism, a phenotype that contributes to group advantage at the cost of disadvantaging itself [42], suggests that a disadvantage at one hierarchical level may be an advantage at another level, justifying why we see this behaviour.

MLSGA [43] uses the idea of multilevel selection by evolving on both an individual level and sub-population level (collectives). Each collective consists of a portion of the entire population, and is evaluated and evolved with collective level selection, just as an individual in the population would be. Coevolutionary mechanics were further included in cMLSGA [2] to allow different evolutionary algorithms for the individuals of different sub-populations, allowing collective coevolution which increases the generality of the algorithm. The sub-populations are groups based on similarities between individuals, allowing specialized roles to be formed. Further, evolution at the population level can be seen as a form of social learning and communication at the sub-population level, where information is transferred between the sub-populations across multiple generations. This means the algorithm incorporates selection, recombination, mutation, specialized roles and social learning at the population level.

### 3.2. Evolution strategies

ES [44] use the mechanics of selection, recombination and mutation to evolve the population. However, unlike traditional GAs, ES are self-adapting with a distinct set of endogenous and exogenous parameters [44]. Endogenous parameters are evolved along with an individual solution and passed down to offspring individuals, independent of others in the population. This contrasts with GAs where all parameters such as crossover and mutation rate are set beforehand, making them all exogenous. Endogenous parameters control the strategy of ES algorithms by changing the statistical properties for mutation. There are two steps of recombination and mutation for an individual *i* in each generation:

— recombination to form the endogenous strategy parameters *s*(*i*)
— recombination to form the solution variables *y*(*i*)
— mutation of the strategy parameters
— mutation of the solution variables using the strategy parameters

CMA-ES [45] is an improvement to the traditional ES algorithm. It uses a covariance matrix to generate its mutation distribution that is adaptive and evolves with the search, allowing the strategy parameters to adapt closely during local search. The parameters are adapted based on statistics gathered through previous generations. Self-adaptive endogenous parameters can be seen as a form of evolvability, in the sense that the capacity to evolve also changes [46]. The mechanism of passing down and evolving an extra set of parameters changes the capacity of evolution in the population, as the mutation rate is changed and adapted based on previous generations. As evolvability is similar to tuning hyperparameters, the use of a set of self-adapting parameters to control mutation automates tuning of the mutation rate. As such it uses selection, recombination and mutation, alongside some elements of evolvability.

### 3.3. Differential evolution

Differential evolution [47] (DE) is another branch of evolutionary computation that is similar to GA. Selection, recombination and mutation operators are all used in DE. The main difference between GAs and DE algorithms is the representation of the individual. DE uses real value vectors and their recombination and mutation operators revolve around the difference between two vectors.

A popular variant to the DE algorithm, the success-history adaptive DE [48] (SHADE) adapts the scaling factor and crossover rate parameters dynamically by changing them based on a memorized history of successful parameters in previous generations. The parameters are changed for each individual, rather than as a whole population. This is similar to social learning where successful parameters from other individuals are used to evolve the parameter selection for new individuals. Additional memory is stored through multiple generations for robustness, so a poor set of parameters in one generation does not negatively impact the rest of the search. As such it uses computational elements to improve selection, recombination and mutation alongside some cultural elements of social learning.

### 3.4. Memetic algorithms

Memetic algorithms [49] are inspired by the human information concept of memes [40] as a simple unit of knowledge that can be transferred and evolved in human society and culture. Memetic algorithms take this concept and apply it onto an evolutionary framework. Genetic algorithm concepts of selection and recombination are used, but instead of mutations to make additional changes to offspring solutions, further local search techniques such as simulated annealing [50] are used to improve the fitness of offspring solutions as much as possible before the cycle is repeated for the next generations. The addition of local search heuristics to improve offspring solutions is more similar to the concepts of evo-devo and phenotypic plasticity than epigenetics or cultural information transfer. Multi-objective memetic algorithms such as M-PAES (memetic-parento archived evolution strategy) [51] borrow the use of a non-dominated archive of solutions, and combine them with local search optimizers. These algorithms use selection and recombination mechanisms with a variety of local search techniques that can be considered to be plastic or evolutionary developmental approaches, but would not be considered epigenetic, as the local search behaviour does not change according to environmental changes. Hybrid memetic algorithms that use PSOs [52] or other swarm intelligence algorithms as their local search method could be considered to include cultural elements of social learning.

### 3.5. Swarm intelligence

In contrast to evolutionary algorithms, swarm intelligence algorithms focus on collective behaviour and the transfer of information across the population. Typically, a single generation is used and the population traverses the search space with different mechanics analogous to different behaviour in animals. Some algorithms also include additional genetic components such as GB-ABC [53], an artificial bee colony (ABC) algorithm based on genetic operators.

#### 3.5.1. Particle swarm optimization

Particle swarm optimization (PSO) is an algorithm developed in 1995 by Eberhart & Kennedy [15] for optimizing nonlinear functions. The algorithm is based on the concept of social behaviour and sharing information. A population of particles represents potential solutions in the search space, at each iteration, each particle updates its velocity and position. The velocity update is based on a combination of the best position found so far by the particle, and the best position found so far by the entire swarm. This is an example of social learning, as the velocity is determined both through the best solution found by an individual, and the best solution found so far by the entire swarm.

The comprehensive learning particle swarm optimizer [54] (CLPSO) extends the idea of social learning. Rather than basing the velocity on the single best solution found so far, CLPSO incorporates all other particles in the swarm during the velocity update to increase diversity. The algorithm was able to show significant improvements to performance, especially to multimodal problems. The PSO is based solely on social learning at the individual level whereas CLPSO extends this to the population level.

A successful PSO algorithm for multi-objective problems is the speed-constrained multi-objective PSO [55] which uses the concepts of Pareto dominance and a nearest neighbour density estimator to select leaders. The velocity of particles is also limited to disallow extreme values. The algorithm is based on social learning like other PSO algorithms, but with an added element of selection to choose leading particles.

#### 3.5.2. Competitive swarm optimizer

The competitive swarm optimizer (CSO) [56] uses pairwise competitions between particles in its population to learn instead of the personal and global best positions in PSO. In each generation, particles are randomly paired and their fitness values compared. The loser is updated by learning from the winner and the winner continues to the next generation without any changes. In this case, half the population is updated while the other half is not updated every generation.

The CSO algorithm has shown fast convergence properties in multi-objective problems [57] where it can find Pareto fronts in fewer generations than other GA and PSO algorithms. However, its performance suffers on multimodal problems due to a lack of diversity.

Despite the drastic changes of CSO compared to PSO algorithms, it is still based on social learning with the addition of a selection mechanism from the pairwise competition.

#### 3.5.3. Ant colony optimization

ACO [16] simulates a population of ants that moves through the search space probabilistically based on pheromone trails left behind by previous generations. Pheromone values decrease with each iteration so that old trails fade and new ones form as the search space is explored. Some pheromone trails will be reinforced if the next generations continue to follow the same path, leading to higher pheromone values, which are associated with better solutions. Although ACO uses multiple iterations/generations, there is no information transfer through genetic inheritance. The individuals in the previous generation leave behind ecological signals with pheromone trails that affect the behaviour of new generations, but there are no genetic operators in use. This can be seen as ecological inheritance, which has been defined here as part of social learning. The use of pheromone trails is core to the concept of ACO. Popular ACO algorithms such as the Max-Min ant system [58] (MMAS) and the continuous orthogonal ant colony [59] (COAC) focus on improving the mechanics of the pheromone trails. MMAS limits the maximum and minimum pheromone values on the trails to avoid stagnation. COAC uses orthogonal design to split up the search space into regions for fast exploration. This was shown to improve the convergence speed for continuous unimodal problems, finding the optimal solution in less than half the number of function evaluations, at the cost of convergence speed for multimodal problems. The ACO is based on social learning at the individual level and specialized roles.

#### 3.5.4. Artificial bee colony

In an ABC [60], the population is split into three different types of bees: employed bees, onlooker bees and scout bees;

— Employed bees search for better food sources in their local neighbourhood and share that information with onlooker bees in the region. They are able to remember new food sources if they are better than an existing food source in its memory.
— Onlooker bees take the information given by employed bees and move towards new food sources based on the information.
— Scout bees search for new food sources randomly without taking into account any information. They become employed bees when a new food source is found.

The three types of bees carry out different roles depending on their proximity to food sources. Social learning can also be observed as the bees change behaviour based on information from other bees and contextual clues from the environment. The role of an individual bee is not static, for example employed bees become scouts when their food source is exhausted. Some variants on the ABC algorithm include genetic operators (GB-ABC [53]) to improve global and local search for binary optimization problems. The ABC algorithm uses both specialized roles and social learning between individuals with the GB-ABC variant including genetic operators.

#### 3.5.5. Grey Wolf Optimizer

The Grey Wolf Optimizer (GWO) [61] is an algorithm inspired by grey wolf social structure and hunting techniques. It mimics a leadership hierarchy with four types of grey wolves, alpha, beta, delta and omega. During a search, the alpha, beta and delta wolves are the three best solutions found so far. All other individuals update their position according to the positions of the alpha, beta and delta wolves. Two coefficient vectors ***A*** and ***C*** are used to fluctuate between exploration, searching for prey, and exploitation, attacking the prey. When |*A*| > 1 the wolves diverge from the prey instead of moving towards it. Similarly, |*C*| > 1 emphasizes attacking the prey so the wolves move faster towards it while |*C*| < 1 de-emphasizes attacking.

There is a use of specialized roles among the wolf pack to form the hierarchy and social learning occurs during a hunt, when the omega wolves follow the alpha, beta and delta wolves for direction. Information exchange between the wolves is used as the omega wolves move to position themselves based on the positions of the wolves higher up in the hierarchy. The GWO algorithm uses both specialized roles and social learning between individuals.

#### 3.5.6. Butterfly optimizer

The butterfly optimizer [62] (BO) aims to simulate the mating behaviour of butterflies. It splits a population of butterfly individuals into two groups, males and auxiliary butterflies, in different specialized roles. The male butterflies operate in either perching or patrolling behaviour for exploitation and exploration of the search space, respectively. Male butterflies learn and follow auxiliary butterflies to better positions as a form of social learning. This allows for faster exploration of the search space, as auxiliary butterflies can continue to explore when some males decide to perch. The attractiveness of a location affects the probability that a male butterfly goes into perching behaviour at that location. This is an environmental factor affecting the individual’s behaviour similar to an epigenetic effect, but because there is only a single generation, such behavioural effects are not passed down and therefore the algorithm is not epigenetic.

An improved variant of butterfly algorithm, effective butterfly optimizer with covariance matrix adapted retreat phase [63] (EBO/CMAR) uses a crossover operator to increase diversity and a retreat phase on a third group of butterflies to improve convergence during local search. After each iteration, information is exchanged between each group of butterflies to replace the worst individuals in one group with the best individuals from another. This is social learning on a population level, as each group of butterflies uses different behaviour and the best individuals from other groups can be learned from. The BO uses both specialized roles and social learning between individuals with the EBO/CMAR using genetic operators to improve the performance.

#### 3.5.7. Firefly algorithm

The FA [64,65] is based on the flashing patterns and behaviour of fireflies. Firefly individuals are attracted to each other proportional to their brightness (fitness) and the distance between two individual fireflies. Dimmer (less fit) individuals then move towards the brighter (more fit) individuals. A firefly’s brightness is determined by the landscape of the objective function, meaning their fitness is related to an individual’s phenotype rather than their genotype. Elements of selection are used, as fireflies are more attracted to bright individuals. Social learning is also observed, as an individual moving towards brighter fireflies becomes brighter themselves.

#### 3.5.8. Cuckoo search

Cuckoo search [66] is an algorithm inspired by the aggressive egg laying behaviour of cuckoo birds, which lay eggs in the nests of other birds. The algorithm uses the concept of Levy flights [67] to create a random walk for the population. Eggs laid in nests represent solutions. In each iteration, there is a probability for the cuckoo eggs to be thrown out or the nest abandoned by the host birds as a selection mechanism. In biological mechanisms, Cuckoo search only uses selection.

Improvements to the algorithm were made by adding either a genetic or cultural component. In Cuckoo-GRN (Cuckoo search with genetically replaced nests) [68], the convergence of the algorithm was improved by replacing abandoned nests with crossover and mutation genetic operators. This leads to faster convergence as new nests were created genetically using existing nests with high fitness. Another variant, modified Cuckoo search (MCS) [69] adds a cultural mechanism instead of a genetic mechanism. When new eggs are generated, information is exchanged between a fraction of eggs with the best fitness. New eggs are then generated in midpoint positions between the two chosen eggs. The additional information sharing also improves convergence compared to the original Cuckoo search. These two variations demonstrate how the addition of simple biological mechanisms helps improve performance of an algorithm.

### 3.6. Summary of current algorithms in a biological framework

**Figure 3.**
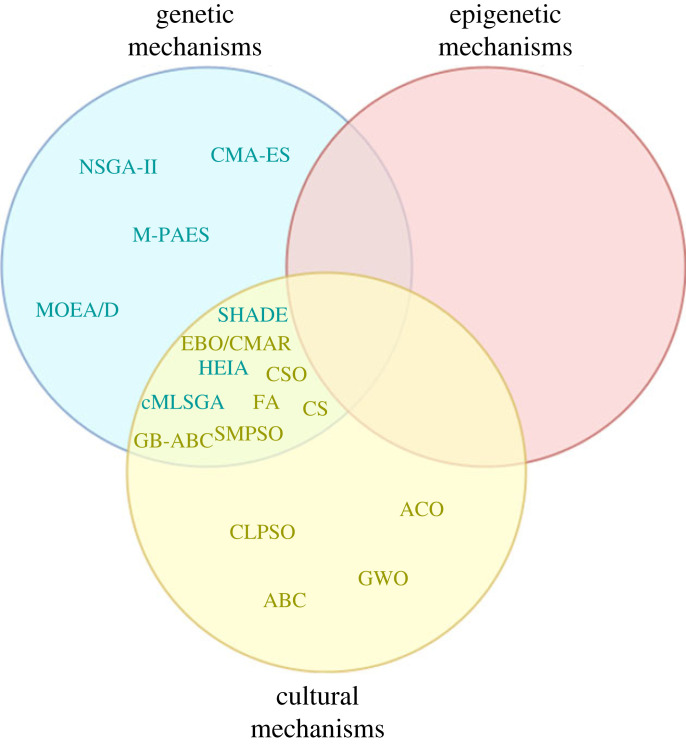
How bioinspired algorithms fit into biological sources for phenotypic variation.

Figure 3 categorizes the mechanisms of the main families of evolutionary algorithms and swarm intelligence under either genetic, epigenetic or cultural information transfer, using a single algorithm to represent each family. In general, most of the categories of algorithm use a single category of information transfer, the evolutionary algorithms focus on different forms of genetic transfer with mechanisms focused around changing the selection of the population to mate and swarm intelligence focuses on different forms of cultural information transfer. Many of the popular evolutionary algorithms such as NSGA-II [32], MOEA/D [34] and SHADE [48] have diverged from their biological roots into mathematical or statistical methods.

However, there are both evolutionary algorithms and swarm intelligence algorithms that show some overlap between genetic and cultural mechanisms. For example, cMLSGA uses multilevel selection by evolving both the individuals and the collectives of individuals, and GB-ABC [53] adds genetic operators to the bee colony algorithm. The mechanisms resulted in increased diversity for cMLSGA and increased convergence for GB-ABC. The main gap is in epigenetic transfer with only EBO/CMAR, using elements of this type of information transfer but which still can’t be considered to be fully epigenetic. There are other algorithms that aim to incorporate epigenetic mechanisms but do not meet the criteria of high citation count or high performance in benchmarks. These algorithms will be discussed next.

## 4. Missing concepts from the extended evolutionary synthesis

While the extended synthesis provides a number of opportunities, not all of the elements provide easy inspiration for practical optimization problems. Evolutionary and developmental biology (Evo-devo) includes the developmental stages of living organisms and the evolution of developmental processes; which in many ways most closely replicate the initial vision proposed by Turing [13] of generating a child and teaching it to learn. Adapting the concept for general evolutionary algorithms would require substantial expansion of the simple genotype model of evolution used in evolutionary algorithms, such as including properties relating to gene regulation, homeobox genes and allometry. Evo-devo provides a number of developmental steps for fine-tuning an organism, but in the algorithmic world this is unnecessary when more generations can be run instead. It is possible to apply evo-devo specific applications, such as digital architectures [70], where domain-specific concepts in architecture can be linked to evo-devo processes, and the time required to generate solutions makes fewer generations more suitable. The design of neural networks using GA [71] mimics evo-devo concepts with limited success as neural networks already learn through their training process, and evolutionary algorithms are typically used to network parameters such as weights and architecture [72]. In Turing’s paper [13], he also suggested the idea of teaching and training neural networks through evolution rather than an evo-devo approach.

Genomic evolution involves the evolution of genome architecture itself. In computational terms, genomic evolution would involve the evolution of the number of variables or range of values in an optimization problem. These values are typically set based on the problem and do not require evolutionary mechanics applied to them.

This leaves epigenetics, niche construction, and phenotypic plasticity as unused mechanisms with the most promise for providing excellent bio-inspiration for new algorithms. Particular focus is placed on the exploration of epigenetic mechanisms, alongside the possible benefits of niche construction and phenotypic plasticity

### 4.1. Epigenetics

Epigenetics plays an important role in adapting a population to new conditions and environments quickly. In computational terms, epigenetic mechanisms should help to improve convergence, potentially spreading changes through a population faster than genetic evolution, and improve stability around a solution in the face of environmental changes. There are a number of different epigenetics processes such as DNA methylation, bookmarking, gene silencing, gene repression and genomic imprinting that are triggered by different factors such as environment, diet or the presence of certain chemical compounds [73].

In the modern synthesis view of evolution, genetic changes occur randomly and fitness is guided by natural selection. Similar to this, the development of modern GA focus on improving the selection process while keeping genetic changes random to retain diversity. A number of generations is required for suitable traits with high fitness to spread throughout a population, even with mechanisms such as elitism. Epigenetic inheritance allows for faster changes based on environmental cues which can occur simultaneously among multiple individuals in the same generation. These adaptive adjustments do not affect the underlying genotype, allowing regular genetic processes to occur and epigenetic processes to be reversed. For example, in GA, the final solution to an optimization problem could be generated from the phenotype consisting of the combination genes and phenotypic changes from the epigenetic tags. This allows solutions or values not found in the genotypes to be in the final solution, allowing rapid changes to not require slower genetic propagation. These rapid changes are ideal for scenarios such as dynamic optimization problems or variable-length problems. The inheritance of epigenetic tags in parallel with genetic inheritance results in continual rapid changes with a diverse set of tags among a population, without disrupting underlying genetic processes [74]. This is an important aspect in evolutionary biology to guide phenotypic variation in a direction suitable for the environment instead of relying solely on random mutation and natural selection.

### 4.2. Niche construction

Niche construction is the concept of organisms altering their environment to better suit their needs, and in doing so leaving behind these useful alterations for the next generations to benefit from [75]. Because this typically requires individuals in the population to change their own environment, it is difficult to accomplish computationally. Optimization problems are usually predefined and the fitness landscape is dependent on the problem. If an algorithm is able to change the fitness landscape to suit the solutions it produces, it changes the problem definition, meaning the solutions found may no longer be applicable or useful to the original problem.

However, the concept of niche construction can be expanded to not only look at the fitness landscape as the environment that can be changed. The social environment in which the methods of interaction between individuals could be changed over time. Improved methods of communication and information flow can also be considered niche construction. In this case, algorithms such as the ACO could be argued as using niche construction through the pheromone trails that change the information pathways between workers.

### 4.3. Phenotypic plasticity

The concept of phenotypic plasticity is the idea that an organism’s behaviour or physiology could change due to environmental factors [76]. Information from the environment is taken and used when creating the final phenotype from the genotype. For example, if a genotype for a neutral network is evolved first through training, a plastic neural network would be one that is able to learn and respond to the environment it is placed in, to alter the final weights of the network, leading to a plastic phenotype after genetic evolution. Plastic neural networks have been explored in the past [77–79] with success, showing the benefits of including phenotypic plasticity in intelligent systems that need to run and adapt in the environments they are placed in. Learning can be further extended as a developmental process to include altering the topology of the network, although this has yet to be explored. The class of optimization problems that focuses on dynamic problems, where the objectives and constraints may be unstable and change throughout the search, would also be suitable for algorithms with flexible, plastic responses. Algorithms in swarm intelligence can exhibit some of this behaviour, for example when new bees take different roles in the artificial bee colony depending on the number of existing bees in other roles. However, there is no scale or range of plastic responses in reaction to a changing environment in the ABC implementation.

Phenotypic plasticity is a relevant concept particularly in intelligent agents that require adaptation in a working environment. While genotypes can be evolved during an optimization search, phenotypic plasticity allows for learning and adaptation after the initial search.

## 5. Opportunities to include epigenetic mechanisms in evolutionary algorithms

### 5.1. Existing studies

The key concept of epigenetics is to allow for fast variation when appropriate. Existing studies [27–29] inspired by epigenetics are currently missing the key feature of triggering mechanisms based on the fitness of the population to the environment. Mechanisms are triggered probabilistically without any distinction between individuals, parents, and how epigenetic marks are passed on. This probabilistic method is more akin to bet hedging [80] than epigenetics, where the mechanics do not improve individual fitness in stationary conditions with no drastic changes in the environment, but create advantages in extreme conditions such as being stuck at a local optima.

An epigenetic algorithm based on intra-generational epigenetic processes used by bio-molecules was developed by Periyasamy [27]. This system mimics cellular organization, with individuals of bio-molecules performing independent tasks in a swarm-like manner, and require specific conditions to be met. The focus on the epigenetic processes uses no genetic operators, which misses a genetic component to contribute to the final phenotype. So while the use of epigenetics can improve the convergence of this algorithm, it potentially loses diversity from the lack of genetics and can get stuck at local optima.

**Figure 4.**
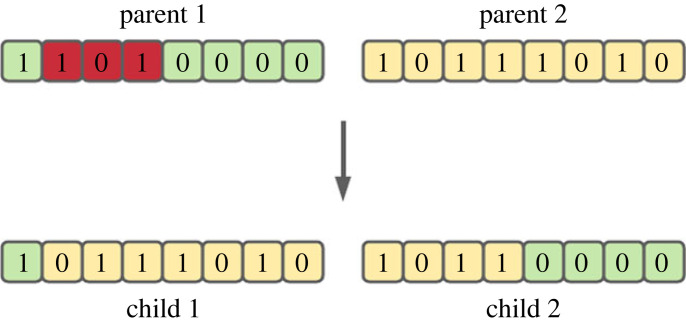
Concept of the cytosine methylation epigenetic process used in [28], where part of the parent genotype is blocked by the epigenetic process.

The epigenetic process of cytosine methylation is incorporated into a genetic algorithm to solve the Knapsack problem [28]. Cytosine methylation blocks a part of an individual’s genotype during the crossover operation. Figure 4 shows the methylation operation used in reproduction, where a portion of the crossover from parent 1 is blocked, transferring a larger portion of the fitter parent’s genotype while silencing the poorer parent. This constant probability for methylation to occur means the epigenetic tags are assumed to always be passed to new generations without any dynamic changes to external factors.

Finally, the concept of methylation and gene silencing is incorporated into an epigenetic algorithm [29]. Similar to [28], a number of parent genes may be masked based on the probability of the epigenetics mechanism occurring. The constant probability of trigger for the epigenetic mark means the marks do not evolve or change through new generations. The occurrence of the epigenetic trait is probabilistic, so it occurs with some chance rather than being triggered due to environmental or parental cues. This misses the concept of allowing fast variation when appropriate. So, while some algorithms are inspired by epigenetics they are currently missing the key features that lead to the benefits seen in evolution. These features are outlined in the following subsections.

### 5.2. Epigenetic tags: the epigenotype

A key aspect not captured by existing studies is the inheritance and transfer of epigenetic information to future generations. While the epigenetic mechanisms implemented were accurate, this transfer is important as it guides the direction of phenotypic change. Without this aspect, the epigenetic mechanisms simply act as another form of mutation, probabilistically switching genes on and off. To include the epigenetic information transfer, epigenetic tags can be used to form an epigenotype [81], to keep a history of inherited epigenetic changes and allow changes to an individual’s genotype to be triggered based on these tags. Epigenetic tags can be added and removed based on signals from the environment, or based on inheritance and crossover operations when forming offspring individuals [82]. Epigenetic tags can also help control gene expression in response to environmental changes. In biology, this helps to form ‘memory’ based on changing environments [83]. The memory of the recent environment allows for fast adaptation and stability. By controlling how genes are expressed using an epigenotype, suitable traits are constantly adjusted to improve fitness before longer term genetic changes can be applied.

**Figure 5.**
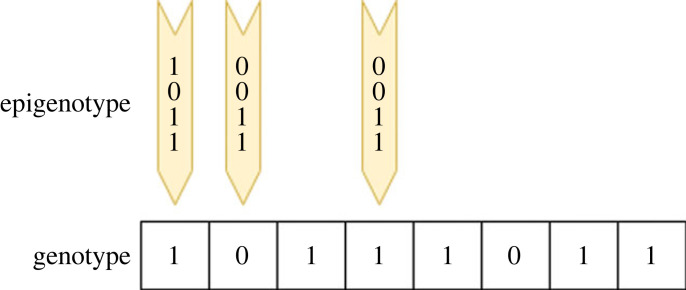
An epigenotype with epigenetic tags for some genes in the genotype.

Computationally, each variable in a solution can contain a set of tags that can be inherited and modified. Figure 5 shows the epigenetic tags on top of some variables in the genotype. The tag can be used to encode mechanisms to alter the variable after genetic operators are applied. These mechanisms can then help increase convergence by guiding phenotypic variation in a direction matching selection pressures. The underlying genetic mechanisms are further unaffected by epigenetic changes, and epigenetic changes can be easily reversed, reducing the cost of poor mutations compared to genetic mutations. The assumption of the one way flow from genotype to phenotype in biology is adopted, where the epigenotype sits over the underlying genotype without a backwards flow of information. It is possible that the underlying genome could benefit from learned changes of the epigenotype. However, this could also affect genes that are suitable for the long term being replaced by short-term epigenetic adaptations, leading to over convergence in a local optima. The advantage of fast adaptations that can be quickly turned on and off could then be lost. It is not known whether a backwards flow of information could provide other benefits, but GA can serve as a useful platform to further investigate the advantages or disadvantages of such backflow.

The epigenotype represents the three key aspects of epigenetics:

— the transfer of epigenetic information,
— self-adaptability to environmental changes,
— fast convergence from direction variation.

These aspects may have potential benefits especially for dynamic problems where the Pareto front is not static. The self-adaptability and fast convergence would allow algorithms with an epigenotype to adjust to a changing Pareto front quickly.

### 5.3. Epigenetic mechanisms

With the use of an epigenotype [81], epigenetic tags can be added, removed and inherited to future generations. An epigenotype alters the phenotype without changes to the underlying phenotype. The tags can then be used to encode different epigenetic mechanisms to be triggered. The mechanisms have a range of effects on the genotype, such as switching genes on and off, or reducing gene expression based on the location and number of tags in the epigenotype.

#### 5.3.1. Genomic imprinting

Genomic imprinting [84] restricts the expression of a gene to one parent. Imprinting is useful when the imprinted alleles lead to different phenotypes that affect an individual’s fitness. This process does not directly change the genotype and can prevent segregation according to Mendel’s laws at the phenotype level. Epigenetic tags are imprinted in the germline and cause the imprinted genes to be expressed from only one parent [85].

There are three hypothesized theories for the process of imprinting

— the kinship theory [86]—The theory suggests that an imbalance exists between parental genes due to conflicting fitness strategies from both parents. This is mostly apparent in sexual reproduction where the father and mother have differing interests to pass on their own genes.
— The sexual antagonism theory [87]—This theory uses sex-specific selection pressure. It predicts an uneven allele frequency between males and females when natural selection favours one sex over the other so that offspring genes are enriched to benefit a particular sex.
— The maternal-offspring coadaptation theory [88]—Based on the correlation between the genes of the mother and the maternal genes of the offspring, the maternal-offspring coadaptation theory states that the offspring is more likely to inherit from its mother because it leads to a higher probability that the offspring has a positive interaction with its maternal phenotype, and the interaction leads to higher fitness.

#### 5.3.2. Gene regulation

The presence of epigenetic tags enables gene regulation mechanics to occur on the tagged genes. There are multiple forms of gene regulation: gene silencing, gene activation and gene repression. All forms of gene regulation affect the expression of the affected genes leading to variation in phenotypes from the same genotype [89]. Gene silencing is a mechanism for turning entire sections of the genotype on and off independent of mutation. In evolutionary biology, gene silencing has the effect of protecting the host organism from viruses [90] by silencing genes that are used in viral reproduction. In terms of convergence and diversity, convergence should be increased and diversity decreased as silenced genes are not fully expressed compared to other genes.

Gene repression acts on individual genes rather than entire sections of the genotype. In evolutionary biology it switches off genes whose products are required to maintain cell functions [91]. To implement this computationally, each variable in a candidate solution can be switched on or off, based on the tags of the epigenotype. The modification of the tags can be based on the fitness of the individual, adapted based on the progress of the search or based on environmental cues. The concept of gene silencing has been shown to provide convergence benefits in dynamic multi-objective problems for MOEA/D [92], where 12 out of 16 test problems (FDA, JY and UDF benchmark problems) showed statistically significant positive improvement compared to the baseline algorithm. The only statistically significant negative result still yielded an improvement for 40% of the duration of the dynamic problem.

## 6. Conclusion

Evolutionary computation is an area that now covers many hundreds of different variants. Despite its biological roots, and the diversity of mechanisms available in nature, these algorithms can broadly be split into two main categories of evolution: evolutionary algorithms, which take inspiration from genetic inheritance, and swarm intelligence algorithms, which take inspiration from cultural inheritance. The reason for this lack of diversity is speculated to be that similar underlying biological mechanisms are often recycled under new banners, making it hard to understand which mechanisms have already been considered. To understand which have been considered, and which have been overlooked, existing evolutionary computation algorithms are categorized under a contemporary biological classification inspired by the extended evolutionary synthesis. It confirms that two main types of information exchange, and therefore behaviours, are used in evolutionary computation. Few algorithms combine these types of information exchange but those that do provide excellent performance.

Opportunities for evolutionary computation with niche construction, phenotypic plasticity and epigenetic mechanisms are identified, all of which are key elements in the way evolution is now described with many underlying mechanisms proposed in the biological literature. A focused discussion on epigenetics shows how epigenetic mechanisms provide a self-adaptive means to quickly converge and stabilize populations in changing environments. A key aspect in inspiring algorithms from epigenetics is flagged as the transfer of epigenetic information to quickly change individual phenotypes, which must be reversible so that they do not alter the genotype. The resulting mechanisms are suitable in situations where there is a changing environment during the optimization process. An evolutionary algorithm benefits from both self-adaptive changes and faster convergence based, particularly in dynamic environments that rapidly fluctuate, but may eventually stabilize, or in scenarios where the number of variables are changing. An early example is given of an algorithm using these approaches to improve performance on dynamic problems.

## Data Availability

This article has no additional data.

## Appendix A. Algorithms chosen

**Table 3.** Evolutionary algorithms were chosen if they had more than 1000 citations on Google Scholar, or showed exceptional performance in a competition or benchmarking paper.
